# New Prognostic Biomarkers and Drug Targets for Skin Cutaneous Melanoma *via* Comprehensive Bioinformatic Analysis and Validation

**DOI:** 10.3389/fonc.2021.745384

**Published:** 2021-10-13

**Authors:** Sitong Zhou, Yuanyuan Han, Jiehua Li, Xiaobing Pi, Jin Lyu, Shijian Xiang, Xinzhu Zhou, Xiaodong Chen, Zhengguang Wang, Ronghua Yang

**Affiliations:** ^1^ Department of Dermatology, The First People’s Hospital of Foshan, Foshan, China; ^2^ Institute of Medical Biology, Chinese Academy of Medical Sciences and Peking Union Medical College, Yunnan Key Laboratory of Vaccine Research and Development on Severe Infectious Diseases, Kunming, China; ^3^ Department of Pathology, The First People’s Hospital of Foshan, Foshan, China; ^4^ Department of Pharmacy, Seventh Affiliated Hospital of Sun Yat-sen University, Shenzhen, China; ^5^ The Second School of Medicine, Wenzhou Medical University, Wenzhou, China; ^6^ Department of Burn Surgery and Skin Regeneration, The First People’s Hospital of Foshan, Foshan, China; ^7^ Department of Orthopedics, The First Affiliated Hospital of China Medical University, Shenyang, China

**Keywords:** cutaneous melanoma, biomarker, WGCNA, bioinformatic analysis, experimental validation

## Abstract

Skin cutaneous melanoma (SKCM) is the most aggressive and fatal type of skin cancer. Its highly heterogeneous features make personalized treatments difficult, so there is an urgent need to identify markers for early diagnosis and therapy. Detailed profiles are useful for assessing malignancy potential and treatment in various cancers. In this study, we constructed a co-expression module using expression data for cutaneous melanoma. A weighted gene co-expression network analysis was used to discover a co-expression gene module for the pathogenesis of this disease, followed by a comprehensive bioinformatics analysis of selected hub genes. A connectivity map (CMap) was used to predict drugs for the treatment of SKCM based on hub genes, and immunohistochemical (IHC) staining was performed to validate the protein levels. After discovering a co-expression gene module for the pathogenesis of this disease, we combined GWAS validation and DEG analysis to identify 10 hub genes in the most relevant module. Survival curves indicated that eight hub genes were significantly and negatively associated with overall survival. A total of eight hub genes were positively correlated with SKCM tumor purity, and 10 hub genes were negatively correlated with the infiltration level of CD4+ T cells and B cells. Methylation levels of seven hub genes in stage 2 SKCM were significantly lower than those in stage 3. We also analyzed the isomer expression levels of 10 hub genes to explore the therapeutic target value of 10 hub genes in terms of alternative splicing (AS). All 10 hub genes had mutations in skin tissue. Furthermore, CMap analysis identified cefamandole, ursolic acid, podophyllotoxin, and Gly-His-Lys as four targeted therapy drugs that may be effective treatments for SKCM. Finally, IHC staining results showed that all 10 molecules were highly expressed in melanoma specimens compared to normal samples. These findings provide new insights into SKCM pathogenesis based on multi-omics profiles of key prognostic biomarkers and drug targets. GPR143 and SLC45A2 may serve as drug targets for immunotherapy and prognostic biomarkers for SKCM. This study identified four drugs with significant potential in treating SKCM patients.

## Introduction

Skin cutaneous melanoma (SKCM) is one of the most aggressive skin cancers, accounting for approximately 80% of skin cancer-related deaths (1). Moreover, most melanoma patients relapse or do not respond to treatments due to toxicity, intrinsic drug resistance, and other reasons not completely understood. The molecular characteristics of SKCM show internal heterogeneity, which is the main obstacle to individualized treatment and the main determinant of drug resistance. Therefore, accurate classification of skin melanomas and identification of molecular markers to identify candidate drug targets remain a top priority. This requires a broad understanding of the heterogeneity at the genomic, transcriptomic, and epigenomic levels (2).

At present, research on the expression module of cutaneous melanoma is scant. Although studies have discovered some important genes and pathways, and diagnosis and treatment of cutaneous melanoma has progressed (3), prognosis for cutaneous melanoma is still very poor (4). Detailed profiles of these key genes at the genomic, transcriptomic, and epigenomic levels are even rarer. Therefore, there is an urgent need to identify new drug targets and detailed profiles of these cancer targets to assess their malignant potential and prognosis.

Weighted gene co-expression network analysis (WGCNA) (5) is a commonly used method to study the complex relationships between genes and phenotypes. Its advantage is that WGCNA converts gene expression data into co-expression modules, offering insights into signal networks that may be responsible for the phenotypic characteristics of interest. It is a comprehensive set of R functions used for the weighted correlation network analysis in all aspects. It is widely used in cancer, genetics, and brain imaging to identify candidate biomarkers (6) or therapeutic cancer drug targets (7). It not only helps to compare the process of differentially expressed genes, but also helps to understand the interaction between genes in different co-expression modules.

The CMap is a large dataset that collects transcriptome changes for a variety of small molecules that have been used in human cancer cell lines in experiments and clinics. It mines these data and identifies bioactive compounds with similar or opposite activities based on pattern matching (https://portals.broadinstitute.org/cmap/). Since most CMap compounds are FDA-approved drugs, these analyses have become valuable tools for understanding the mechanism of drug action and drug reuse in pan-cancer studies.

This study aimed to construct a co-expression module using the expression data of cutaneous melanoma. A specific co-expression gene module for the pathogenesis of this disease has been discovered. Combined with GWAS, DEG analysis, and GO/KEGG/GSEA enrichment analysis on the modules of interest and determining the hub genes in each module will help us to understand the potential mechanisms of the genes in these modules. We also conducted tumor immune infiltration analysis, gene DNA methylation, patient survival analysis, isoform expression analysis, and gene mutation analysis of 10 selected hub genes. CMap was used to identify targeted therapy drugs that may be effective treatments for SKCM.

## Materials and Methods

### Data Sources

The two datasets used in this project were an RNA array GSE15605 dataset (8–13) from the GEO database and SKCM RNA-seq data from the TCGA database (https://tcga-data.nci.nih.gov/tcga/). The GSE42352 dataset consisted of 16 normal skin tissues (GSM390208-GSM390223) and 46 primary skin melanoma tissues (GSM390224-GSM390269) in the SKCM group. The platform for GSE15605 was the GPL570. The number of patients in the TCGA-SKCM dataset was 470, including 77 in stage I, 140 in stage II, 172 in stage III, and 24 in stage IV.

### WGCNA Analysis

We used WGCNA to identify the co-expressed gene modules. First, we calculated the Pearson correlation coefficient (PCC) for all the paired genes, and an adjacency matrix was constructed using a power function. The power of *β* was set to 7 (scale-free R2 = 0.9) to ensure a scale-free network. We then converted the adjacency matrix into a topological overlap matrix (TOM) so that the genes with similar expression profiles were clustered into modules using the average-linkage hierarchical clustering method. The top 5,000 coding genes were selected. Notably, the minimum base number of each gene network module was set to 30 in this study. According to the TOM-based dissimilarity, the genes were finally divided into 16 different modules.

### PPI Network Construction

We obtained protein interactions (score > 0.4) of encoding genes using the STRING database (14) (https://string-db.org/), and the interaction network was visualized using Cytoscape (15). The top 10 key genes were obtained using CytoHubba, and the interaction network was drawn.

### Identification of Differentially Expressed Genes

For the GSE15605 dataset, we obtained the gene expression matrix file and the annotation file of the corresponding chip platform (GPL570) simultaneously. The probe signal was converted to the expression value of each gene. If multiple probes corresponded to the same gene, the average value was considered as the final gene signal value. We used the limma (16) package to analyze the DEGs, and the screening criteria were set as |log2 (fold change) |> 2 and *p* < 0.05.

### Functional Enrichment Analysis

We used the metascape database (17) (https://metascape.org/) to perform GO function and KEGG pathway enrichment analysis on the DEGs, with the parameters set as minimum overlap = 3, adjusted *p*-value cutoff = 0.01, and minimum enrichment = 1.5. We found enriched GO terms and KEGG pathways in these DEGs, and *p*-values were adjusted using Benjamini–Hochberg (BH) correction.

### Survival Curve

We used GEPIA 2 (18) to analyze the correlation between the expression levels of the hub genes and survival time of the SKCM patients. According to the best division of the gene expression, SKCM patients were divided into high- and low-expression groups. Kaplan–Meier (KM) survival curves were drawn to represent the survival differences among patients with different gene expression levels.

### Tissue-Specific Expression of Genes and Analysis of Tumor Immune Infiltration

We obtained gene expression profiles from the Expression Atlas (19) (https://www.ebi.ac.uk/gxa/home). We analyzed the gene expression data of the SKCM samples in the TCGA database using TIMER2.0 (20) and determined the correlation between gene expression levels and tumor purity. We also measured the levels of immune cell infiltration.

### DNA Methylation, Isoform Expression, and Gene Mutation Analysis

We obtained information on the level of DNA methylation in the promoter region of genes from UALCAN (21) (http://ualcan.path.uab.edu/index.html). Expression levels of gene isoforms were also analyzed using GEPIA 2. We obtained mutation information for the skin tissue of the original site of the hub gene from COSMIC (https://cancer.sanger.ac.uk/cosmic). The enhancer mutations were obtained using the CancerEnD database (22) (https://webs.iiitd.edu.in/raghava/cancerend/index.html).

### Screening of Small-Molecule Therapeutic Drugs

The selected hub genes were used for potential drug prediction in the CMap. CMap is the most comprehensive transcriptome database for drug intervention and is usually used to explore potential drugs for disease treatment (https://portals.broadinstitute.org/cmap/). A negative connectivity score was considered a potential therapeutic drug. Therefore, enrichment < 0.8 and *p* < 0.01 were used as screening criteria. The PubChem (https://pubchem.ncbi.nlm.nih.gov/) database was used to determine the molecular structure of the identified drugs.

### Patient Tissue Specimens

Twenty-five melanoma specimens and 10 normal skin tissues (**Supplementary Table S1**) were collected between 2015 and 2021. Patient-informed consent was obtained and approved by the First People’s Hospital of the Foshan Subject Review Board.

### Immunohistochemistry Staining

Paraffin-embedded tissues were sectioned at 4 μm for IHC analysis. Antigens were retrieved by incubating the samples in citrate buffer (pH 6.0) for 15 min at 100°C in a microwave oven and naturally cooled to room temperature. After blocking with a mixture of methanol and 0.75% hydrogen peroxide, sections were incubated overnight with appropriate dilatation of primary antibodies (MLANA, Sangon Biotech, 1:100; PMEL, Sangon Biotech,1:100; EDNRB, Sangon Biotech, 1:150; MIA, Sangon Biotech,1:300; GPR143, Sigma, 1:500; SOX10, Cell Signaling Technology, 1:150; PRAME, Cell Signaling Technology, 1:300; TYR, Sangon Biotech, 1:50; MITF, Sangon Biotech 1:80; SLC45A2, Proteintech, 1:150) followed by incubation with a secondary antibody conjugated with HRP (goat anti-rabbit, 1:500, Cell Signaling Technology; goat anti-mouse, 1:800, Abcam). The sections were washed three times with PBS and incubated with AEC (ZSGB-BIO). The analysis process was described in our previous paper (23).

## Results

### Weighted Co-Expression Network Construction and Trait-Related Module Identification

**Figure 1:**
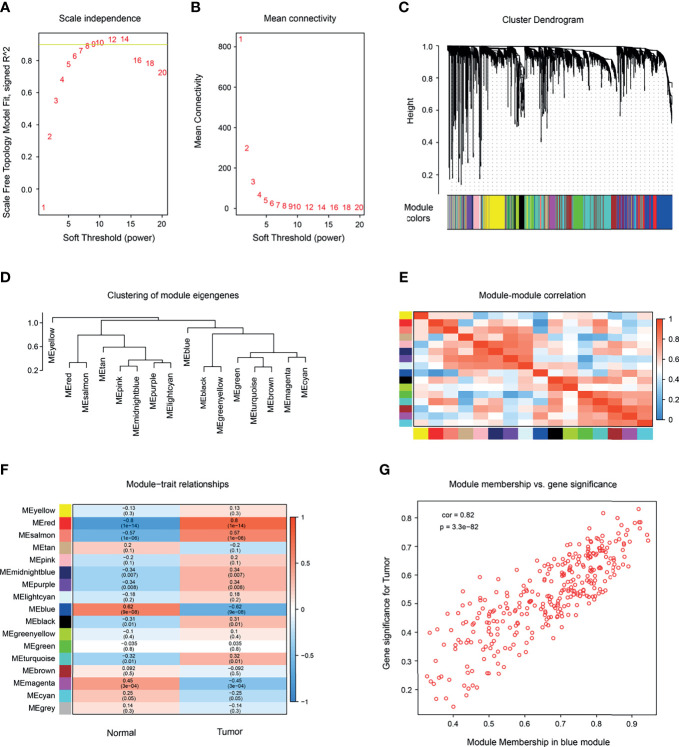
Construction of a co-expression network by WGCNA. **(A)** Determination of soft-thresholding power in WGCNA analysis. Scale-free fit index of WGCNA for various soft-thresholding powers (*β*). **(B)** Analysis of the mean connectivity under the different soft-thresholding powers. **(C)** Dendrogram of the differentially expressed genes (DEGs), clustered based on dissimilarity measure clustering (1-Tom). **(D)** Clustering of module genes. **(E)** Heatmap of the correlation among modules (red represents high correlation; blue represents low correlation). **(F)** Correlation between gene modules and clinical characteristics of melanoma (red indicates high positive correlation; blue indicates high negative correlation). **(G)** The relationship between gene significance and module membership of the blue module was analyzed.

To explore the relationship between SKCM occurrence and gene expression, we conducted WGCNA analysis on GSE15605 and found 16 modules. A total of 50,000 genes were included in the WGCNA analysis. A power of *β* = 7 (scale-free R2 = 0.85) was selected as the soft-thresholding parameter to conduct a scale-free network (**Figures 1A, B**). The selected samples were clustered using the average linkage hierarchical clustering method. A total of 16 modules were identified by clustering (**Figures 1C–E**). Further analysis showed that the red module (**Figures 1F, G**) had the greatest positive correlation with the occurrence of SKCM. There were 333 genes in this module, all of which were protein-coding genes. The red module was selected to identify the hub genes.

### Identification of SKCM-Related Genes by WGCNA and Validation by GWAS

**Figure 2:**
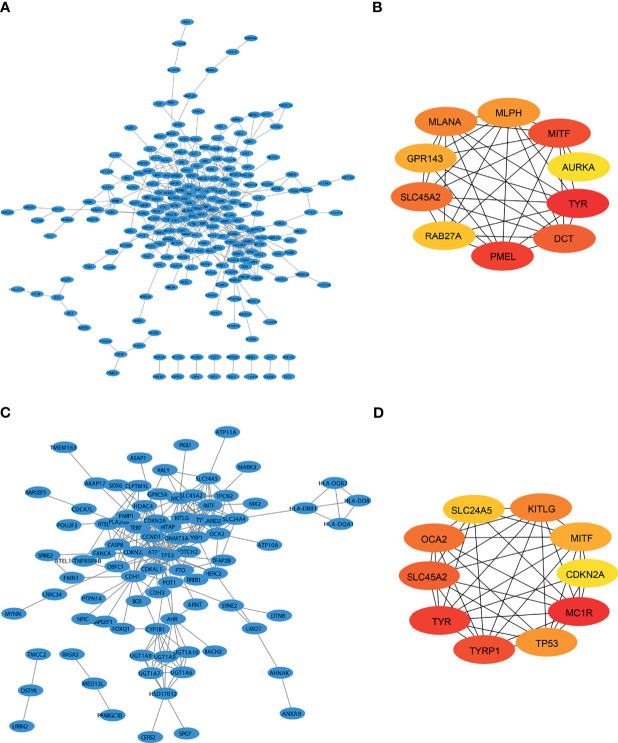
Identification of SKCM-related genes by both WGCNA and GWAS. **(A)** Protein interaction (PPI) network of the genes that are most positively related to SKCM identified by WGCNA. **(B)** PPI network of the top 10 genes identified by WGCNA. **(C)** PPI of SKCM-related protein coding genes identified by genome-wide association study (GWAS). **(D)** The protein interactions of the top 10 genes identified by GWAS.

There were many interactions among the proteins encoded by the 333 genes in the red module (**Figure 2A**), including TYR, PMEL, MITF, DCT, SLC45A2, MLANA, MLPH, GPR143, Rab27A, and AURKA, which were the key components in the PPI network (**Figure 2B** and **Table 1**). In addition, a total of 348 SKCM-related genes were identified using GWAS (https://www.ebi.ac.uk/gwas/). The proteins encoded by these genes had many interactions as well (**Figure 2C**), among which the top 10 key components were MC1R, TYR, TYRP1, SLC45A2, OCA2, KITLG, TP53, MITF, SLC24A5, and CDKN2A (**Figure 2D** and **Table 2**). Among them, TYR, MITF, and SLC45A2 were the common hub genes of the PPI network in both WGCNA and GWAS, which supported the results of WGCNA.

**Table 1:** Information of hub genes related to SKCM identified by WGCNA.

Gene name	Chr	Start (bp)	End (bp)	Strand	Description
SLC45A2	5	33946602	33956490	–	Solute carrier family 45 member 2
DCT	13	94436811	94479682	–	Dopachrome tautomerase
MLANA	9	5890889	5910606	+	Melan-A
AURKA	20	56369389	56392337	–	Aurora kinase A
GPR143	X	9725346	9786297	–	G protein-coupled receptor 143
RAB27A	15	55202966	55319113	–	RAB27A, member RAS oncogene family
PMEL	12	55954105	55973317	–	Premelanosome protein
MITF	3	69739464	69968336	+	Melanocyte inducing transcription factor
TYR	11	89177875	89295759	+	Tyrosinase
MLPH	2	237485428	237555322	+	Melanophilin

**Table 2:** Information of hub genes related to SKCM identified by GWAS.

Gene name	Chr	Start (bp)	End (bp)	Strand	Description
SLC45A2	5	33946602	33956490	–	Solute carrier family 45 member 2
OCA2	15	27871154	28099370	–	OCA2 melanosomal transmembrane protein
TYRP1	9	12685439	12710285	+	Tyrosinase related protein 1
CDKN2A	9	21967752	21995301	–	Cyclin dependent kinase inhibitor 2A
SLC24A5	15	48120990	48142672	+	Solute carrier family 24 member 5
MC1R	16	89912119	89920973	+	Melanocortin 1 receptor
KITLG	12	88492793	88580851	–	KIT ligand
MITF	3	69739464	69968336	+	Melanocyte inducing transcription factor
TYR	11	89177875	89295759	+	Tyrosinase
TP53	17	7661779	7687538	–	Tumor protein p53

### Analysis of SKCM-Related Differentially Expressed Genes

**Figure 3:**
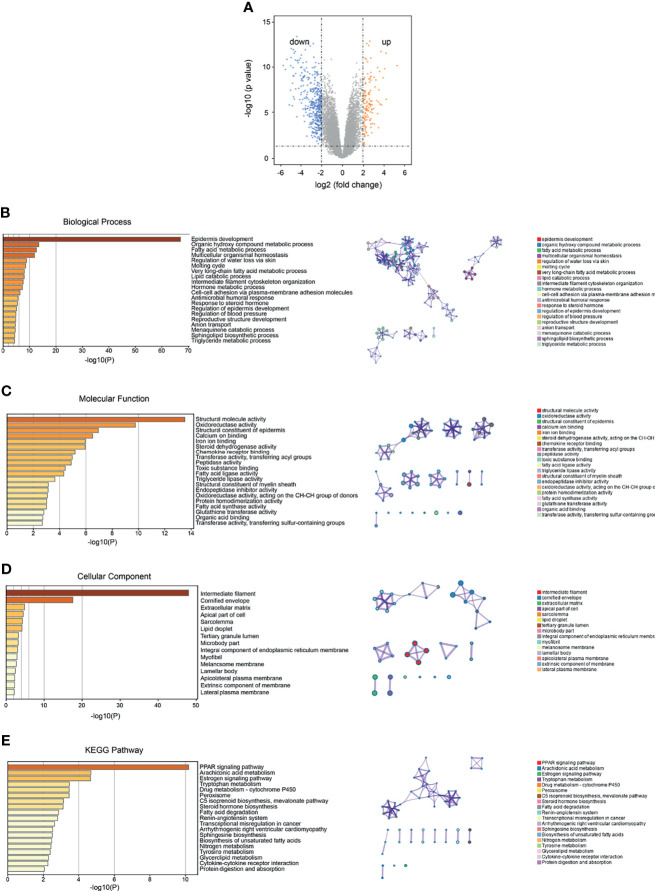
Identification and functional enrichment analysis of SKCM DEGs. **(A)** Volcano plot of the identified DEGs (orange and blue dots represent upregulated and downregulated genes, respectively). **(B)** Functional enrichment analysis of GO (gene ontology) and biological process (BP) of DEGs. **(C)** Enrichment analysis of GO molecular function (MF) of DEGs. **(D)** Functional enrichment analysis of GO cell components (CC) of DEGs. **(E)** Functional enrichment analysis of KEGG (Kyoto Encyclopedia of Genes and Genomes) pathway in DEGs.

Through the analysis of SKCM-related DEGs, we identified 126 upregulated genes and 416 downregulated genes (*p* < 0.05) (**Figure 3A** and **Table 3**). We selectively identified the top significant GO biological process terms and the top significant KEGG pathways meeting the selection criteria (**Supplementary Table S2**). The DEGs showed significant metabolism-related and hormone signaling-related GO terms and KEGG pathways after BH correction. Through GO function enrichment analysis, we found that these genes were mostly enriched in biological processes such as epidermis development, organic hydroxy compound metabolic processes, and fatty acid metabolic processes (**Figure 3B**). The molecular functions are mostly involved in structural molecule activity, oxidoreductase activity, and structural constituents of the epidermis (**Figure 3C**). The related cellular components include intermediate fibers, keratin membranes, and extracellular matrix (**Figure 3D**). KEGG pathway enrichment analysis suggested that these genes were enriched in the PPAR signaling pathway, arachidonic acid metabolism, and the estrogen signaling pathway (**Figure 3E** and **Supplementary Table S2**).

**Table 3:** DEGs of SKCM (top 10 upregulation and top 10 downregulation according to *p*-value).

Gene name	Chr	Start	End	Strand	Description	LogFC	*p*-value
EDNRB	13	77895481	77975529	–	Endothelin receptor type B	2.66285171	1.25E-13
CRACD	4	56049073	56328625	+	Capping protein inhibiting regulator of actin dynamics	2.37323301	2.36E-13
C4orf48	4	2041993	2043970	+	Chromosome 4 open reading frame 48	2.54917458	3.99E-13
SLC45A2	5	33946602	33956490	–	Solute carrier family 45 member 2	3.72836758	1.77E-12
TUBB4A	19	6494319	6502848	–	Tubulin beta 4A class Iva	4.22450924	2.74E-12
SOX10	22	37970686	37987422	–	SRY-box transcription factor 10	2.76028069	9.39E-12
BACE2	21	41167801	41282530	+	Beta-secretase 2	2.2186336	1.00E-11
TRIB2	2	12716889	12742734	+	Tribbles pseudokinase 2	2.32263761	1.31E-11
PLOD3	7	101205977	101218420	–	Procollagen-lysine,2-oxoglutarate 5-dioxygenase 3	2.44516108	2.44E-11
PRAME	22	22556806	22568466	–	PRAME nuclear receptor transcriptional regulator	5.29935881	6.57E-11
TSPAN8	12	71125085	71441898	–	Tetraspanin 8	−4.4020815	3.94E-14
SLC27A6	5	128538013	129033642	+	Solute carrier family 27 member 6	−3.3370382	2.36E-13
MFSD4A-AS1	1	205554272	205569305	–	MFSD4A antisense RNA 1	−2.803971	2.36E-13
DCD	12	54644589	54648493	–	Dermcidin	−7.7019636	2.80E-13
PIP	7	143132077	143139739	+	Prolactin induced protein	−6.7613591	3.99E-13
HMGCS2	1	119748002	119768905	–	3-hydroxy-3-methylglutaryl-CoA synthase 2	−3.0959742	4.45E-13
SCGB2A1	11	62208673	62213943	+	Secretoglobin family 2A member 1	−4.9284683	8.57E-13
RERGL	12	18080869	18320107	–	RERG like	−3.5612684	1.12E-12
KRTAP4-6	17	41139433	41140487	–	Keratin associated protein 4-6	−4.7315792	1.12E-12
KRTAP4-9	17	41105332	41106488	+	Keratin associated protein 4-9	−4.5516704	1.77E-12

### Combined Analysis of WGCNA and DEGs, and PPI Network of Intersectional Genes

**Figure 4:**
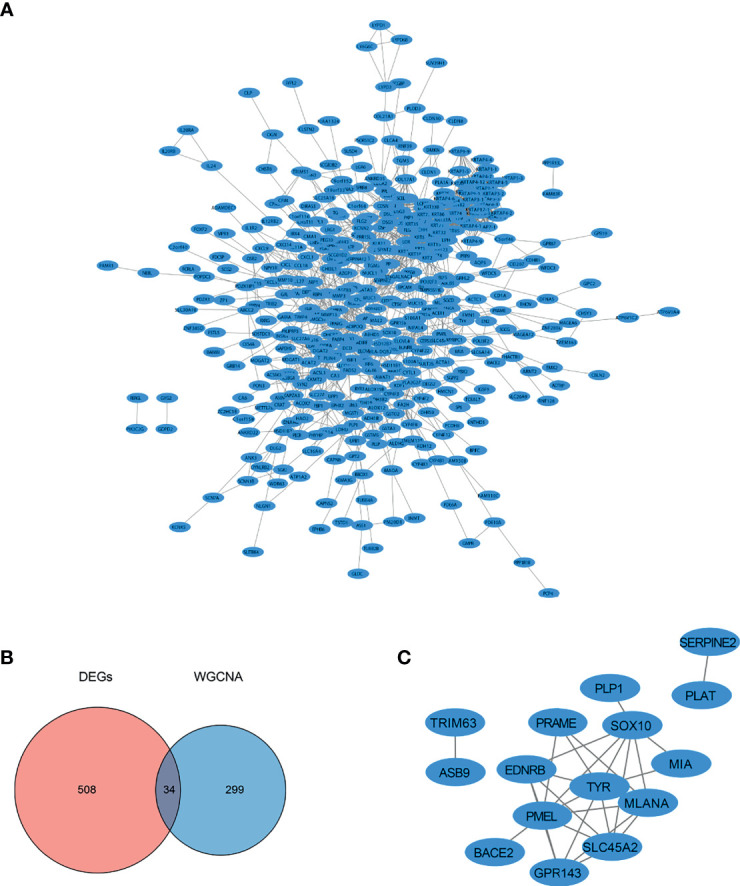
PPI network diagram of the DEGs. **(A)** PPI network of the genes obtained from DEGs analysis. **(B)** Intersection of DEGs identified by the DEGs analysis and SKCM-related genes identified by WGCNA. **(C)** PPI network of intersecting DEGs and SKCM-related genes identified by WGCNA.

The STRING database (https://string-db.org/) was used to analyze the PPI interactions among the DEGs, and a total of 421 nodes and 1,743 protein pairs were obtained with a combined weight score > 0.4. The interaction network was visualized using Cytoscape (3) (**Figure 4A**). The intersection of DEGs and SKCM-related genes identified by WGCNA included 34 common genes (**Figure 4B**). Based on the ranking of the key genes at the intersection of DEGs and WGCNA, we obtained seven key genes, which did not include TYR, MITF, and SLC45A2. To date, we have screened and identified 10 hub genes, including MLANA, PMEL, EDNRB, MIA, GPR143, SOX10, PRAME, TYR, MITF, and SLC45A2 (**Figure 4C**). The 10 selected hub genes were used for further analysis.

### Expression of Hub Genes in Different Tissue of Human

We first analyzed the expression of the 10 hub genes in normal human tissues. From the data of GTEx and 32 Uhlen’s lab, genes were highly expressed in skin tissues and are provided in **Supplementary Figure S1A**, and genes were also expressed in other tissues and are listed in **Supplementary Figure S1B**.

### Ten Hub Genes as Prognostic Markers of SKCM

**Figure 5:**
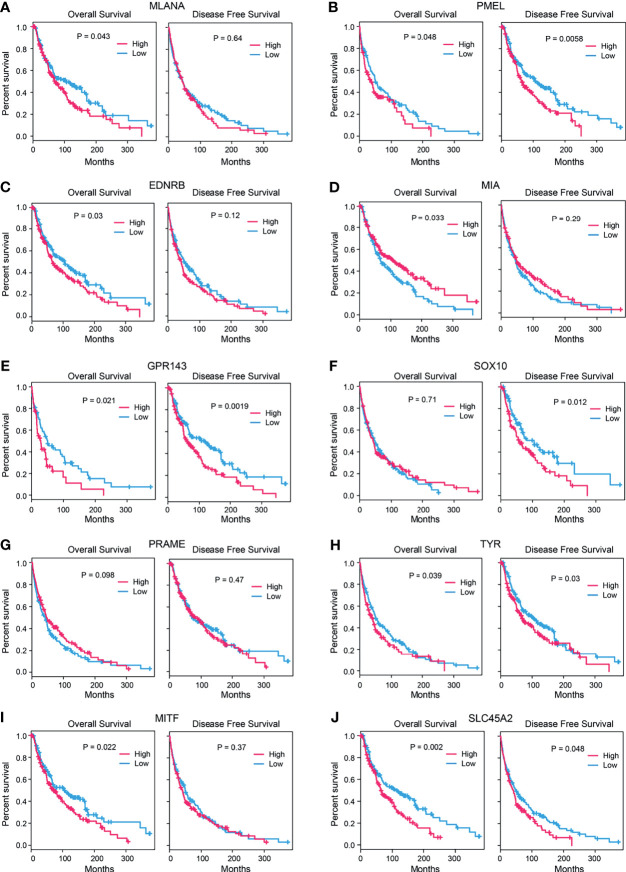
Survival analysis of candidate genes. Correlation between the expression levels of candidate genes **(A)** *MLANA*, **(B)**. *PMEL*, **(C)** *EDNRB*, **(D)** *MIA*, **(E)** *GPR143*, **(F)** *SOX10*, **(G)** *PRAME*, **(H)** *TYR*, **(I)** *MITF*, and **(J)** *SLC45A2* and the survival rates (left: overall survival, right: disease-free survival) of the SKCM patients (*p*-value: log-rank test).

Combined with the clinical data, we analyzed the survival rate of these 10 hub genes, including the overall survival (OS) rate and disease-free survival (DFS) rate. We found that, except for SOX10 and PRAME, the remaining eight genes were significantly associated with the OS of patients (*p* < 0.05). Moreover, gene expression levels were negatively associated with the OS of patients (*p* < 0.05). The expression trend of the MIA gene was opposite since the expression trend of MIA was positively associated with the OS of patients. The expression levels of PMEL, GPR143, SOX10, TYR, and SLC45A2 were significantly correlated with DFS (*p* < 0.05), and the higher the gene expression level, the lower the DFS rate of patients. There was no significant correlation between the other five genes (**Figure 5**).

### Abnormal Expression of Immune Cells and Hub Genes Exhibits Correlation to Immune Microenvironment of SKCM

**Figure 6:**
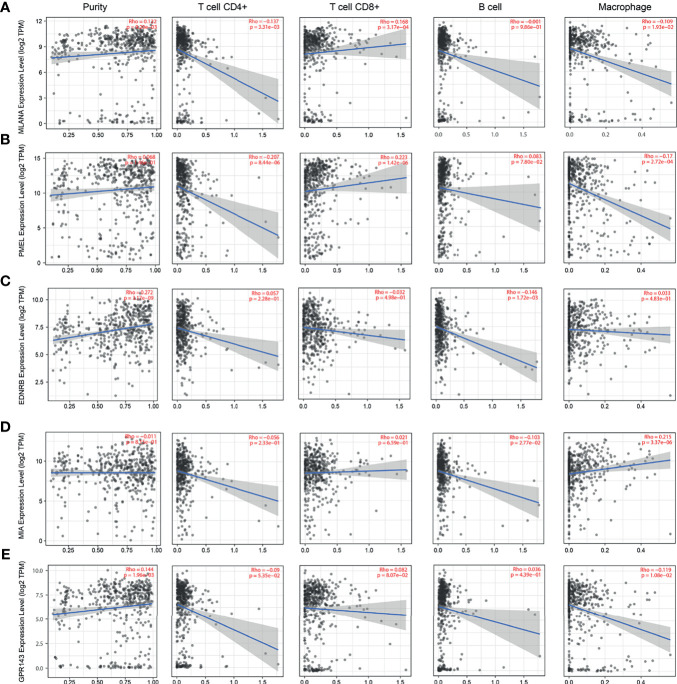
Correlation between candidate genes and immune cell infiltration of *MLANA*, *PMEL*, *EDNRB*, *MIA*, and *GPR143*. **(A)** Correlation between the expression of candidate genes **(A)** *MLANA*, **(B)**. *PMEL*, **(C)** *EDNRB*, **(D)** *MIA*, and **(E)** *GPR143* and the levels of immune cell infiltration (tumor purity, CD4+ T cells, CD8+ T cells, B cells, and macrophages from left to right, respectively).

**Figure 7:**
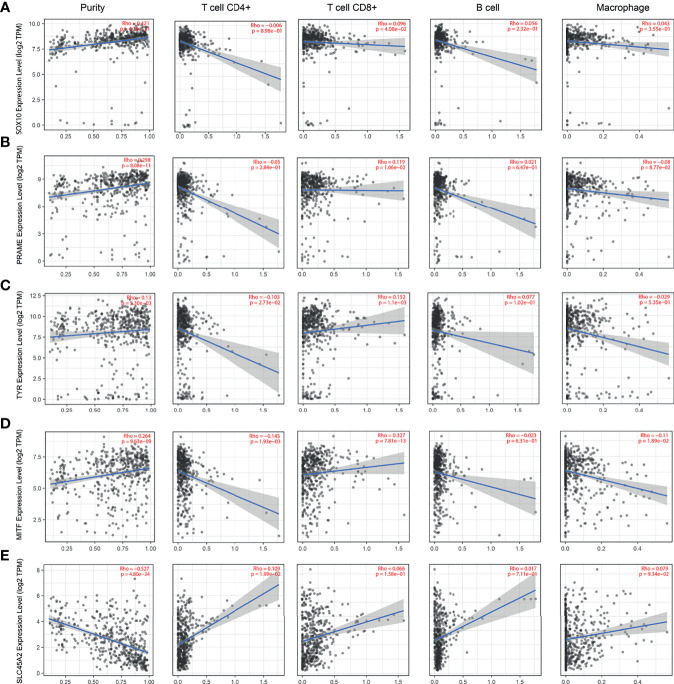
Correlation between the candidate genes and immune cell infiltration of SOX10, PRAME, TYR, MITF, and SLC45A2. The correlation between the expression of A–E candidate genes: **(A)** *SOX10*, **(B)** *PRAME*, **(C)** *TYR*, **(D)** *MITF*, and **(E)** *SLC45A2* and the infiltration level of immune cells (tumor purity, CD4+ T cells, CD8+ T cells, B cells, and macrophages from left to right, respectively).

The lack of sufficient SKCM-related datasets, including clinical data, fomented our use of the TCGA database for subsequent analysis. First, we analyzed the tumor immune infiltration of these 10 hub genes. The results showed that their expression levels were positively correlated with SKCM tumor purity (except PMEL, MIA, and SLC45A2). The expression of these genes was negatively correlated with the infiltration level of CD4+ T cells and B cells, but not with the infiltration level of CD8+ cells and macrophages (**Figures 6** and **7**). It is worth noting that the expression pattern of SLC45A2 is slightly different from other genes.

### DNA Methylation of Hub Genes

Next, we explored the relationship between DNA methylation in the promoter region of hub genes and the occurrence of SKCM to elucidate the potential mechanisms of abnormal upregulation of these hub genes. By comparison, except for GPR143, SOX10, PRAME, and MITF, the methylation levels of other genes in stage 2 SKCM were lower than those in SKCM stage 3 (*p* < 0.05; **Supplementary Figure S2**).

### Isomer Expression Analysis of 10 Hub Genes

We simultaneously analyzed the isomer expression level of the 10 hub genes. MLANA, MLANA-001, MLANA-003, and MLANA-004 were highly expressed in the SKCM (**Supplementary Figure S3A**). The expression levels of PMEL-002, PMEL-004, PMEL-005, and PMEL-017 were higher in SKCM (**Supplementary Figure S3B**). EDNRB, EDNRB-001, EDNRB-003, and EDNRB-004 were highly expressed in SKCM (**Supplementary Figure S3C**). MIA-001, MIA-003, and MIA-004 were highly expressed in SKCM (**Supplementary Figure S3D**). GPR143, GPR143-001, GPR143-002, and GPR143-004 were highly expressed in SKCM (**Supplementary Figure S3E**). SOX10, SOX10-002, and SOX10-004 were highly expressed in the SKCM (**Supplementary Figure S3F**). PRAME, PRAME-001, PRAME-003, and PRAME-201 were highly expressed in SKCM (**Supplementary Figure S3G**). For TYR, the expression level of the TYR-001 isomer in SKCM was higher (**Supplementary Figure S3H**). MITF, MITF-001, MITF-004, MITF-005, and MITF-201 were highly expressed in the SKCM (**Supplementary Figure S3I**). SLC45A2, SLC45A2-001, and SLC45A2-002 were highly expressed in the SKCM (**Supplementary Figure S3J**).

### Mutation Analysis of Hub Genes

Among these 10 hub genes, we found that all 10 hub genes had gene mutations in the skin tissue (**Supplementary Table S3**). However, no mutations in the gene enhancer region associated with SKCM were found (**Supplementary Table S4**).

### Small-Molecule Therapeutic Drugs

**Figure 8:**
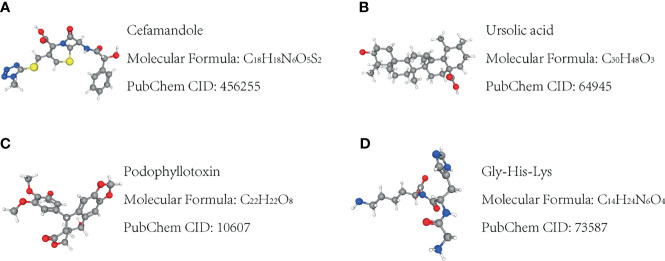
Molecular structure of the potential small-molecule drugs predicted by CMap for the treatment of SKCM based on the 10 hub target genes. **(A–D)** Molecular structure of the four targeted drugs.

Using the CMap database, the 10 hub genes were analyzed to predict potential therapeutic drugs for SKCM. A total of four drugs, cefamandole, ursolic acid, podophyllotoxin, and Gly-His-Lys, were identified (**Table 4** and **Figure 8**). These drugs have potential inhibitory effects on the 10 hub genes.

**Table 4:** Ten hub genes were used to predict potential drugs for the treatment of SKCM.

Cmap name	Mean	*N*	Enrichment	*p*	Specificity	Percent non-null
Cefamandole	−0.76	4	−0.861	0.00068	0	100
Ursolic acid	−0.739	4	−0.852	0.00092	0	100
Podophyllotoxin	−0.696	4	−0.85	0.00093	0.0588	100
Gly-His-Lys	−0.726	3	−0.842	0.00791	0.0448	100

### Histologic analysis

**Figure 9:**
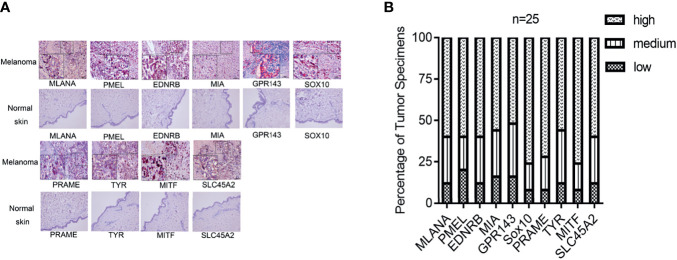
Gene expression of the hub genes in melanoma tissue and normal skin specimens. Using IHC staining, all the hub genes (*MLANA, PMEL, EDNRB, MIA, GPR143, SOX10, PRAME, TYR, MITF*, and *SLC45A2*) are expressed at higher levels in the melanoma tissue (*n* = 25) when compared to normal skin. **(A)** IHC staining. **(B)** Quantification of the protein levels of the hub genes in melanoma tissues. IHC: Immunohistochemistry. IHC stain, AEC, original magnification: 100 × (inset, IHC stain, AEC, original magnification: 400 ×).

We next explored the protein expression levels of hub genes in melanoma tissues and normal skin. The IHC staining results showed that all 10 molecules (MLANA, PMEL, EDNRB, MIA, GPR143, SOX10, PRAME, TYR, MITF, and SLC45A2) showed higher expression levels (*n* = 25) than normal skin (*n* = 10) (**Figure 9**).

## Discussion

Melanomas are highly heterogeneous at the genetic, expression, and epigenetic levels. The rapid progress in understanding this heterogeneity is making molecular classification and individualized treatment of melanoma possible. Despite considerable progress, early recognition of invasive melanoma remains a goal in the field of melanoma research. The underlying co-expression modules that drive heterogeneity among patients, including key biomarkers and therapeutic drug targets, remain unclear. Furthermore, biomarkers take various forms, including DNA methylation, isomer expression, and genetic mutations in cancer cells. Therefore, in this study, we comprehensively identified 8 of 10 hub genes, which proved to be independent prognostic factors for SKCM. In addition, eight of these genes seem to be closely related to immune cell infiltration and tumor purity of SKCM. We further illustrated a detailed profile of methylation levels, isomer expression levels, and mutations in these selected hub genes.

WGCNA was used to build a co-expression network, revealing a red module composed of genes that are significantly related to the clinical characteristics of SKCM patients. Among the 10 hub genes screened by WGCNA, three genes (TYR, MITF, and SLC45A2) were also SKCM-related when screened by GWAS. This result demonstrated that the method of screening SKCM-related hub genes using the WGCNA method is stable and reliable. Next, we analyzed the intersection genes of WGCNA and DEGs and identified seven key genes (MLANA, PMEL, EDNRB, MIA, GPR143, SOX10, and PRAME), except TYR, MITF, and SLC45A2.

Many studies have reported that some of the 10 hub genes are SKCM-related, which function in tumorigenesis and malignant phenotypes such as MLANA, PMEL, EDNRB, MIA, SOX10, PRAME, TYR, and MITF. However, few reports implicate GPR143 and SLC45A2 in SKCM. MITF is a well-known melanoma-related transcription factor. EDNRB, MITF, and TYR are melanogenesis-related genes. EDNRB and MITF also belong to the “pathways in cancer” pathway. In melanoma, the BRAF (V600E)/ERK1/2 pathway is especially involved in regulating the expression and/or activity of MITF, suggesting the role of MITF as a melanoma addiction oncogene. MITF is considered a driving factor of melanoma progression, but its role in inhibiting invasion and metastasis has also been confirmed. Therefore, it is important to better understand the intracellular mechanisms of MITF (24). EDNRB, a receptor of the endothelin signaling pathway, is essential for the development of neural crest melanocytes and is associated with the progression of melanoma. EDNRB was found to be upregulated in melanoma metastasis and altered tumor–host interactions leading to melanoma progression (25). TYR is a mouse gene encoding tyrosinase, which triggers the first and rate-limiting step in melanin biosynthesis. MITF and GPR143 were expressed at higher levels in tumors from non-responders to DTIC/TMZ therapy (26). MLANA/MART1 was reported to be transcriptionally regulated by MITF in melanocytes and melanomas (27). PMEL is a co-expression gene with BAP1 (BAP1 loss is common in uveal melanoma UM and is associated with a worse prognosis). In both CM and UM, PMEL encodes a melanosome structural protein (28). Serum MIA interacts with extracellular matrix proteins, and its overexpression is also observed in breast cancer and colorectal cancer (29). MIA is also a reliable tumor marker in the serum of patients with malignant melanoma (30). SOX10 is a transcription factor that positively regulates MITF expression in melanocytes (31). Previous data suggest that SOX10 is an important melanocyte marker. Gene expression profiles of different stages of melanoma progression show that PRAME is expressed in primary melanoma, but not in healthy skin tissues or benign melanocytic lesions (nevi or moles), indicating that PRAME expression may be an event in melanocytic transformation (32). PRAME was significantly associated with an increased risk of metastasis in UM, and PRAME also had prognostic value in UM (33). Previous GWAS studies have identified MITF, TYR, and SLC45A2 as SKCM susceptibility-related genes (34). Although they are associated with individual risk estimation, a thorough understanding of these biomarkers based on patient survival analysis, tumor immune infiltration analysis, gene DNA methylation, isoform expression analysis, and gene mutation analyses is rare. Therefore, a comprehensive understanding of risk genes may be more meaningful.

Furthermore, we screened two novel biomarkers, GPR143 and SLC45A2. GPR143 is a gene related to X-linked ocular albinism type 1. GPR143 is a protein-coding gene expressed only in pigment cells. It has been proven that GPR143 is closely related to SKCM occurrence and development. One study demonstrated that GPR143 was the most highly upregulated GPCR in SKCM and suggested that GPCR mRNA signatures characterize specific tumor types (35). SLC45A2 encodes a putative transporter that is mainly expressed in the pigment cells. SLC45A2 mutation leads to oculocutaneous albinism type 4 (OCA4). The polymorphism of SLC45A2 is associated with variation in pigmentation (36). However, the roles of GPR143 and SLC45A2 in SKCM development remain unclear. To our knowledge, only a few studies have reported the potential impact of GPR143 and SLC45A2 on the prognosis of SKCM. We determined that GPR143 and SLC45A2 were not only significantly upregulated in SKCM tissues, but were positively correlated with worse prognosis, suggesting important contributions to the pathogenesis of SKCM. Furthermore, comprehensive analyses showed that both genes appeared to be promising candidates as therapeutic drug targets and prognostic predictors.

From the data of GTEx and 32 Uhlen’s lab, we found that these genes were highly expressed in skin tissues, and these genes were also expressed in other tissues. Survival curves indicated that eight hub genes (MLANA, PMEL, EDNRB, MIA, GPR143, TYR, MITF, and SLC45A2) were negatively associated with the OS of patients (*p* < 0.05), except for SOX10 and PRAME. The expression levels of PMEL, GPR143, SOX10, TYR, and SLC45A2 were also significantly correlated with DFS. Therefore, these were all independent predictors of SKCM. Functional enrichment analysis of SKCM-related DEGs revealed significant metabolism-related, hormone signaling-related GO terms and KEGG pathways. SKCM is known to be associated with melanogenesis and pigmentation caused by UVB-induced α-MSH/MC1R pathways. α-MSH-stimulating hormones can subsequently alter metabolic pathways and reactions (37). The enrichment results suggested that hub genes selected from these DEGs may play key roles in SKCM and may be therapeutic drug targets and potential prognostic indicators of SKCM.

The 10 genes were also confirmed to be associated with immune cell infiltration using the TIMER algorithm. The results showed that the expression levels of MLANA, EDNRB, GPR143, SOX10, PRAME, TYR, MITF, and SLC45A2 were positively correlated with SKCM tumor purity (except PMEL, MIA, and SLC45A2). The expression of the 10 genes was negatively correlated with the infiltration level of CD4+ T cells and B cells, but not with the infiltration level of CD8+ cells and macrophages. The tumor environment consists of tumor cells, stromal cells, and tumor-infiltrating immune cells of both innate and adaptive lineages (38). The composition of tumor-infiltrating immune cells varies with cancer type (39). Tumor-infiltrating T cells show the phenotype and functional characteristics of exhausted T cells (40), indicating that they are impaired due to tumor antigen overload and various tumor immune escape mechanisms. T cells (CD3+, CD8+, and CD4+) and B cells (CD20+) are associated with better patient outcomes (41); however, the 10 hub genes may be related to worse outcomes caused by the low density of immune T cells.

By using hub genes to predict potential drugs for disease treatment, four drugs were recognized, and previous related research supported that cefamandole, ursolic acid, podophyllotoxin, and Gly-His-Lys can induce cancer apoptosis *in vitro*. Finally, we further validated protein expression levels of the hub genes in our melanoma tissue samples. Additionally, experimental assays demonstrated that all hub genes showed high expression levels in clinical samples.

This study has some notable limitations. First, the sample size was relatively small. Hence, further studies with larger sample sizes and prospective designs are warranted to increase the statistical power and achieve more meaningful outcomes. Second, cross-validation with external validation in future studies on the immune microenvironment of SKCM would be needed to support these conclusions. Third, although microarray-based bioinformatics analysis is a powerful tool for effectively understanding the molecular mechanism of SKCM and identifying potential biomarkers, further experimental verification of these hub genes is needed at the molecular, cellular, and *in vivo* levels.

## Conclusion

We identified and screened 10 genes with prognostic ability for SKCM by combining WGCNA, GWAS, and DEG analysis. These genes are associated with immune cell infiltration in patients with SKCM. Importantly, we further identified these hub genes as independent prognostic factors associated with OS and DFS in patients with SKCM. In addition, we analyzed 10 hub genes at the genetic, transcriptional, and methylation levels. We identified cefamandole, ursolic acid, podophyllotoxin, and Gly-His-Lys, as having anti-tumor functions *in vitro*. Furthermore, we validated the protein expression levels in the SKCM samples. These findings suggest an important prognostic and predictive role for these 10 hub genes in SKCM. This has implications for melanoma immunobiology, and the potential development of multi-omics features to predict survival and response to drug treatment.

## Data Availability Statement

The datasets presented in this study can be found in online repositories. The names of the repository/repositories and accession number(s) can be found at: https://www.ncbi.nlm.nih.gov/gds/?term=GSE42352.

## Ethics Statement

The studies involving human participants were reviewed and approved by the ethics committee of The First People’s Hospital of Foshan. The patients/participants provided their written informed consent to participate in this study.

## Author Contributions

XC, ZW, and RY conceived and designed the study. SZ and YH conducted most of the experiments, data analysis, and wrote the manuscript. JHL, XP, JL, SX, and XZ participated in collecting data and helped to draft the manuscript. All authors contributed to the article and approved the submitted version.

## Funding

This study was supported by the National Natural Science Foundation of China (Nos. 82002913 and 81772136), Foundation of Foshan City (Nos. FS0AA-KJ218-1301-0034 and 2018AB003411), the Special Fund of the Foshan Summit plan (Nos. 2019C002, 2019D008, 2019A006, and 2020A015), the GuangDong Basic and Applied Basic Research Foundation (2021A1515011453), and Yunnan Fundamental Research Projects (202001AT070145).

## Conflict of Interest

The authors declare that the research was conducted in the absence of any commercial or financial relationships that could be construed as a potential conflict of interest.

## Publisher’s Note

All claims expressed in this article are solely those of the authors and do not necessarily represent those of their affiliated organizations, or those of the publisher, the editors and the reviewers. Any product that may be evaluated in this article, or claim that may be made by its manufacturer, is not guaranteed or endorsed by the publisher.

## Supplementary Material

Supplementary Figure S1 Candidate gene expression levels in tissue samples. **(A)** Comparison of the expression levels of candidate genes in various human tissues (data from GTXE). **(B)** Comparison of the expression levels of candidate genes in human tissues (data from 32 Uhlen’s lab).

Supplementary Figure S2 Comparison of the methylation levels in the promoter regions of the candidate genes, MLANA **(A)**, PMEL **(B)**, EDNRB **(C)**, MIA **(D)**, GPR143 **(E)**, SOX10 **(F)**, PRAME **(G)**, TYR **(H)**, MITF **(I)**, and SLC45A2 **(J)**, at the different stages of SKCM. (*p*-value: *t*-test).

Supplementary Figure S3 Expression of the isoforms in the SKCM samples. Comparison of the expression levels of the isomers of the candidate genes, MLANA **(A)**, PMEL **(B)**, EDNRB **(C)**, MIA **(D)**, GPR143 **(E)**, SOX10 **(F)**, PRAME **(G)**, TYR **(H)**, MITF **(I)**, SLC45A2 **(J)**, in SKCM tissues.

Supplementary Table S1 Patient characteristics.

Supplementary Table S2 Functional enrichment analysis of GO and KEGG pathways in DEGs.

Supplementary Table S3 Mutations and related information of the candidate genes.

Supplementary Table S4 Mutations in the enhancer regions of the candidate genes.
