# Supplementary figures and images for: New Prognostic Biomarkers and Drug Targets for Skin Cutaneous Melanoma via Comprehensive Bioinformatic Analysis and Validation
Source: Front Oncol. 2021 Oct 13;11:745384. doi: 10.3389/fonc.2021.745384 (PMC8548670; doi:10.3389/fonc.2021.745384)

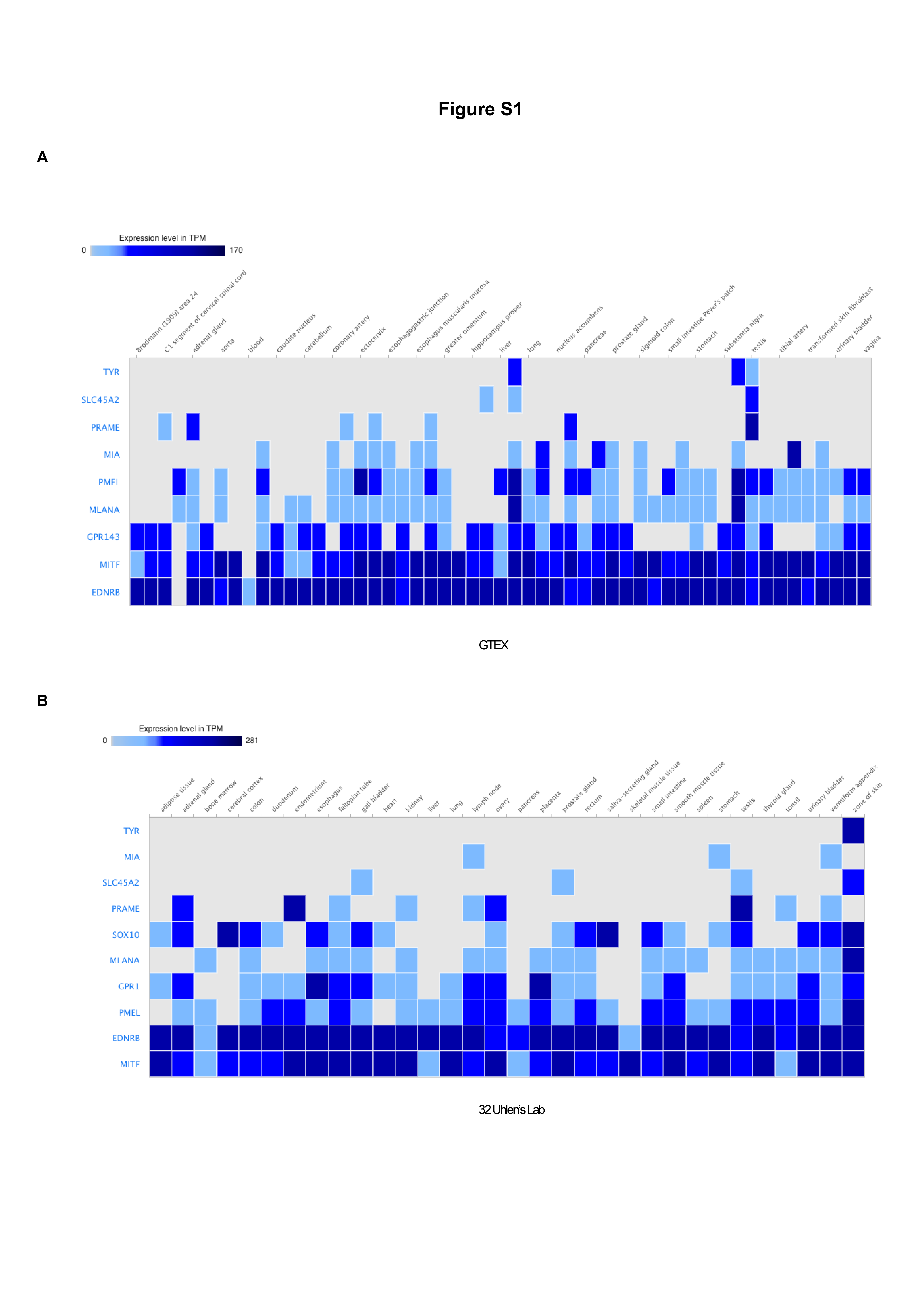

Supplement: Supplementary Figure S1 — Candidate gene expression levels in tissue samples. (A) Comparison of the expression levels of candidate genes in various human tissues (data from GTXE). (B) Comparison of the expression levels of candidate genes in human tissues (data from 32 Uhlen’s lab). [file Image_1.tif]

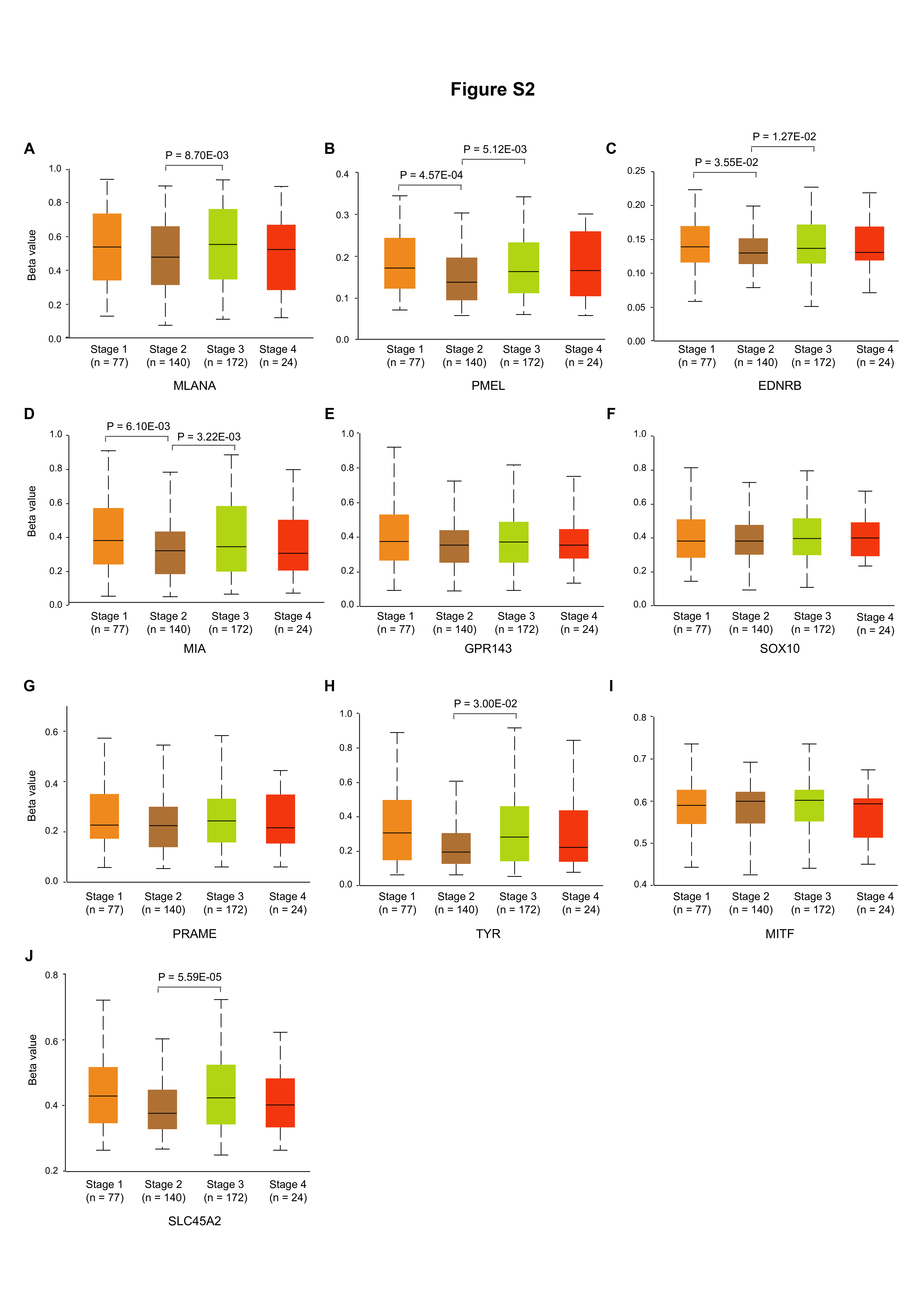

Supplement: Supplementary Figure S2 — Comparison of the methylation levels in the promoter regions of the candidate genes, MLANA (A), PMEL (B), EDNRB (C), MIA (D), GPR143 (E), SOX10 (F), PRAME (G), TYR (H), MITF (I), and SLC45A2 (J), at the different stages of SKCM. (p-value: t-test). [file Image_2.tif]

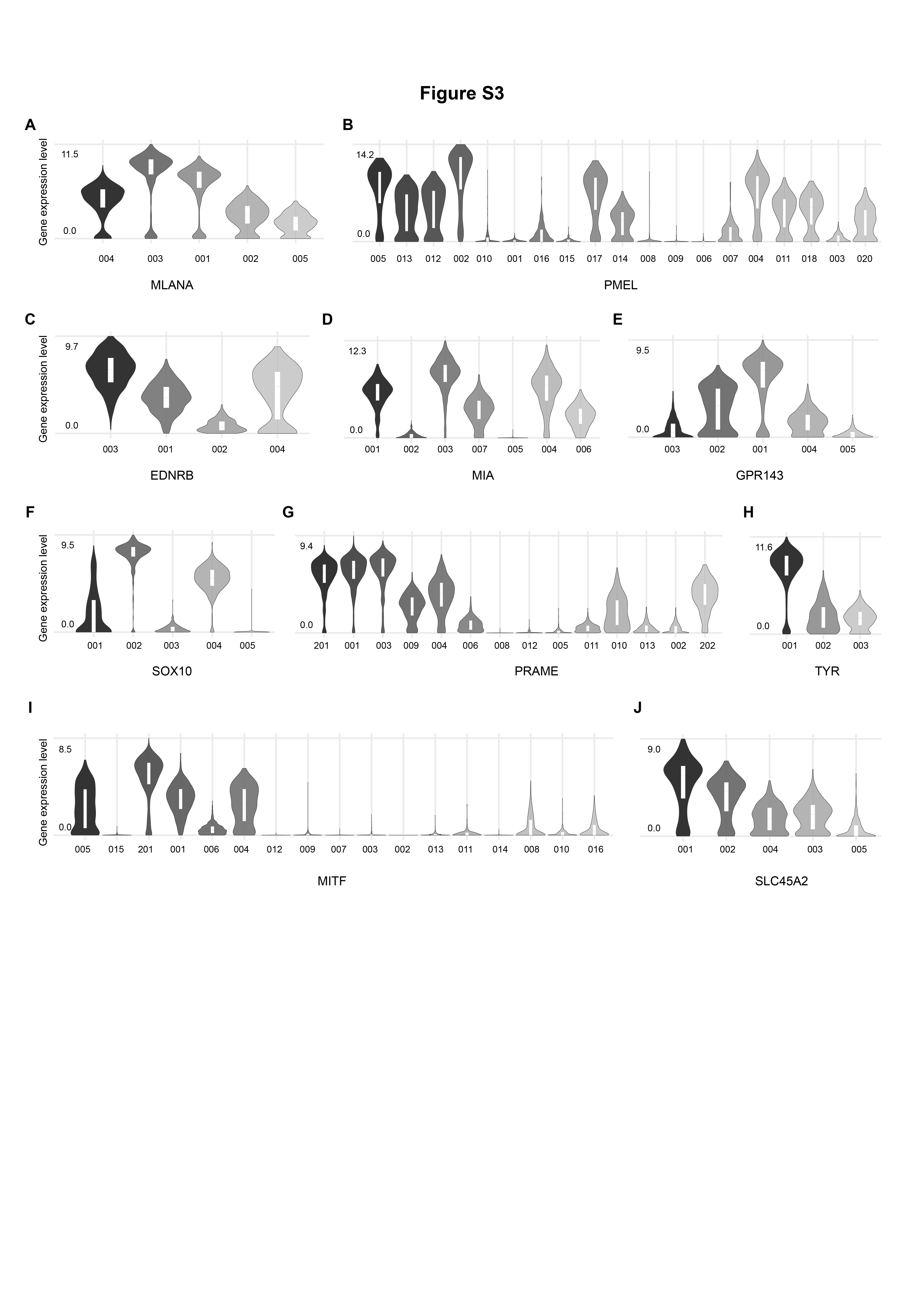

Supplement: Supplementary Figure S3 — Expression of the isoforms in the SKCM samples. Comparison of the expression levels of the isomers of the candidate genes, MLANA (A), PMEL (B), EDNRB (C), MIA (D), GPR143 (E), SOX10 (F), PRAME (G), TYR (H), MITF (I), SLC45A2 (J), in SKCM tissues. [file Image_3.tif]
