# Supplementary material for: New Prognostic Biomarkers and Drug Targets for Skin Cutaneous Melanoma via Comprehensive Bioinformatic Analysis and Validation
Source: Front Oncol. 2021 Oct 13;11:745384. doi: 10.3389/fonc.2021.745384 (PMC8548670; doi:10.3389/fonc.2021.745384)
Supplement: Supplementary Table S1 — Patient characteristics. [file Table_1.docx]

**Table S1. Clinical Characteristics of Melanoma Patients**

| ID | Sex | Age,y | Location | Primary/Metastasis |
| --- | --- | --- | --- | --- |
| Case1 | M | 54 | Right plantar | Primary |
| Case2 | F | 35 | Left plantar | Primary |
| Case3 | M | 82 | Left plantar | Primary |
| Case4 | F | 39 | Right ankle | Primary |
| Case5 | M | 62 | Left plantar | Metastasis |
| Case6 | M | 44 | Left plantar | Metastasis |
| Case7 | M | 55 | Right plantar | Metastasis |
| Case8 | F | 57 | Right plantar | Metastasis |
| Case9 | F | 48 | Face | Primary |
| Case10 | M | 73 | Left plantar | Primary |
| Case11 | M | 64 | Left plantar | Metastasis |
| Case12 | M | 65 | Right plantar | Primary |
| Case13 | M | 66 | Right plantar | Metastasis |
| Case14 | F | 58 | Right plantar | Primary |
| Case15 | F | 64 | Right plantar | Primary |
| Case16 | F | 74 | Right arm | Primary |
| Case17 | M | 66 | Right plantar | Metastasis |
| Case18 | M | 57 | Right plantar | Primary |
| Case19 | M | 52 | Back | Primary |
| Case20 | M | 63 | Left plantar | Metastasis |
| Case21 | F | 74 | Right plantar | Metastasis |
| Case22 | F | 71 | Left ankle | Primary |
| Case23 | M | 63 | Left plantar | Primary |
| Case24 | M | 41 | Left arm | Metastasis |
| Case25 | M | 52 | Head | Metastasis |

F, female; M, male.
